# Noninvasive Estimation of Pulmonary Vascular Resistance Using Right Ventricular Outflow Doppler Analysis

**DOI:** 10.1016/j.jacadv.2025.102079

**Published:** 2025-08-18

**Authors:** Luis Afonso, Chandra Kanth Ala, Aditya Sood, Oluwole Adegbala, Farima Kahe, John Dawdy, Kendall Bell, Abdul Rasheed Bahar, Nimrod Blank

**Affiliations:** a Department of Medicine, Division of Cardiology, Wayne State University, Detroit, Michigan, USA; b Department of Medicine, Wayne State University, Detroit, Michigan, USA

**Keywords:** echocardiography, precapillary, postcapillary pulmonary hypertension, pulmonary impedance, Wood units

## Abstract

**Background:**

Right heart catheterization (RHC) is the gold standard for measuring pulmonary vascular resistance (PVR). Current noninvasive methods lack accuracy, especially in moderate to severe pulmonary arterial hypertension.

**Objectives:**

The objective of the study was to develop and validate a novel echocardiographic method for estimating PVR using right ventricular outflow tract Doppler notch analysis.

**Methods:**

In this prospective study, of 95 patients undergoing RHC, 35 with isolated postcapillary pulmonary hypertension (PVR <2.0 WU) and no discernible Doppler notches were excluded. Of the remaining, 54 patients with interpretable Dopplers were divided into derivation (n = 30) and validation (n = 24) cohorts, all undergoing invasive PVR (iPVR) measurement and echocardiography within 24 hours. Right ventricular outflow tract Doppler, specifically the ratio of notch time (NT) to ejection time, was analyzed to derive a regression equation for non-invasive PVR (niPVR). The model was validated against iPVR and compared with 5 existing methods.

**Results:**

Stepwise linear regression identified the inverse of NT as the strongest predictor of iPVR. After adjusting for heart rate, the model (R^2^ = 0.93) yielded non-iPVR = 7.1∗(ejection time/NT) − 9.36. Validation against 5 established methods showed superior performance across a PVR range of 2.3 to 14.2 WU, with the highest Pearson correlation (r: 0.76; *P* < 0.0001; 95% CI: 0.52-0.89). Bland-Altman analysis confirmed superior agreement.

**Conclusions:**

This novel echocardiographic method offers feasible, rapid, reliable PVR estimation with strong correlation to invasive measurements. Although not a substitute for RHC, this promising preliminary finding from a selective cohort may aid in identifying candidates for invasive testing and guide pulmonary hypertension management. Larger, multicenter validation is warranted.

Pulmonary hypertension (PH) is defined by a mean pulmonary artery pressure (mPAP) >20 mm Hg at rest on right heart catheterization (RHC). Pulmonary arterial hypertension (PAH) describes a subset of patients with PH, characterized hemodynamically by end-expiratory mean pulmonary artery (PA) wedge pressure ≤15 mm Hg and pulmonary vascular resistance (PVR) > 2.0 WU (precapillary pulmonary hypertension).^1^ PVR estimates help differentiate between patients with PH who have a pure precapillary component (PVR >2 WU), combined postcapillary and precapillary PH and those who do not (PVR ≤2 WU-isolated postcapillary PH [IpcPH]).^1^

Although invasive assessment of PVR (iPVR) remains the reference gold standard, accurate non-iPVR (niPVR) estimates are desirable to reduce health care costs, exposure to radiation, need for serial RHC follow-up and related complications.^2^ Numerous echocardiographic methods to estimate PVR exist in the literature.^3^, ^4^, ^5^, ^6^, ^7^, ^8^, ^9^ Most prediction models incorporate a surrogate measure of peak PA systolic pressure, such as tricuspid regurgitation peak velocity (in the numerator) and forward pulmonary flow, such as right ventricular outflow tract (RVOT) Doppler velocity time integrals (VTIs) (in the denominator); derived regression equations are then correlated against iPVR.^3^^,^^4^ Although these methods show modest correlations with iPVR at the low end of the PVR spectrum and clinical utility in certain subsets of patients,^10^^,^^11^ their predictive ability appears to decline with increasing PVR,^7^, ^8^, ^9^^,^^12^ ostensibly related to the curvilinear or nonlinear relationship between tricuspid regurgitation (TR) velocity/PAP, seen with disease progression (increasing PVR).

PAH leads systolic pulmonary valve motion and blood flow perturbations^12^^,^^13^ manifested by notching of the spectral Doppler envelope of the (RVOT).^14^^,^^15^ Systolic Doppler notching is a frequent finding in patients with PAH, in contrast to minimal or no distortion of the envelope in patients with pulmonary venous (postcapillary) hypertension.^13^^,^^16^^,^^17^ Notching represents an abrupt deceleration of systolic flow from reflected tidal waves generated in the setting of increasing pulmonary impedance. The presence of mid-systolic notching (MSN) and late-systolic notching (LSN) in PAH, have been semiquantitatively correlated to both disease severity^13^ as well as prognosis.^13^^,^^18^ We recently described the predictive utility of early systolic notching (ESN) in a selected cohort of patients with computed tomography–confirmed submassive and massive pulmonary embolism.^19^ Based on these aggregate observations, we hypothesized that the timing and location of the notch in the RVOT Doppler envelope could potentially reveal important insight into right ventricular afterload and right ventricular–pulmonary arterial coupling. We further hypothesized that notching would occur temporally earlier in systole commensurate with the elevation in pulmonary vascular impedance. We leveraged this information to derive a regression equation incorporating relevant RVOT Doppler notch characteristics in our prediction model to estimate PVR noninvasively.

In this prospective study, we sought to: 1) develop and test a prediction model to quantitatively determine PVR noninvasively in patients with known or suspected PH; and 2) as a secondary objective, we sought to compare the performance of our model with other methodologies reported in the literature in the same cohort of patients using iPVR as the gold standard.

## Methods

This study protocol was approved by the Wayne State University Institutional Review Board. Patients with known or suspected PH referred for RHC with transthoracic echocardiograms obtained within 24 hours of the RHC were included. Exclusion criteria included atrial fibrillation, interval administration of pulmonary vasodilators, loop diuretic agents, intravenous inotropes, or mechanical ventilation between invasive and noninvasive assessments. Patients with PH were classified according to the 2022 European Society of Cardiology/European Respiratory Society guidelines for the diagnosis and treatment of PH.^1^ The subgroups of PH studied included isolated precapillary PH and those with combined precapillary and postcapillary hypertension, as both groups exhibit RVOT Doppler notching (PVR >2.0 WU), in contrast to those with IpcPH (no RVOT Doppler notching, iPVR <2.0 WU), sequestered from our final analyses.

RHC was performed using the standard protocol using a Swan-Ganz catheter (5-F or 7-F, Baxter Healthcare, Edwards Critical Care Division). Cardiac output was determined using the assumed Fick method. Oxygen consumption (indexed Vo_2_) was assumed to be 125 mL/m^2^ body surface area based on Dehmer formula as per lab protocol.^20^ Supplemental oxygen was not administered unless the patient used chronic supplemental oxygen at rest, in which case the same dose was administered throughout the RHC to maintain a steady state. Pressures obtained included right atrial pressure, right ventricular (RV) systolic pressure, PA systolic and diastolic pressure, mPAP, and pulmonary capillary wedge pressure (PCWP). The wedged position of the tip of the Swan-Ganz catheter during the recording of the occlusion waveform (PCWP) was verified fluoroscopically. Both automated Mac-Lab (GE HealthCare) pressure measurements (averaged over a few respiratory cycles) and manual end-expiratory measurements were collected, and the data were analyzed only with end-expiratory pressures which is the preferred optimal way of obtaining RHC pressures. Mean PAP was obtained by using the standard formula: mPAP = PA diastolic pressure (PADP) + 1/3 (PA systolic pressure − PADP).

Invasive PVR, expressed in WU, was estimated using the equation: iPVR = mPAP−mean PCWP/cardiac output.^10^^,^^11^

### Imaging

Echocardiographic variables for all patients were analyzed from transthoracic echocardiograms obtained with the Philips Cx-50, and EPIQ (Philips Medical Systems) or the GE E9 or E95 Ultrasound (GE Medical Systems) systems. After optimal alignment, Doppler interrogation of the RVOT was performed from the parasternal short-axis view at the level of the aortic valve or from the subcostal short-axis view, with the sample volume placed approximately 0.3 to 0.5 cm proximal to the pulmonic valve. Machine gain, filters, reject, and scale settings were adjusted to precisely define the onset and the end of the systolic RVOT Doppler profile. RVOT Doppler velocity-time integral (VTI) was obtained in the standard manner by tracing the systolic RVOT pulse wave (PW) Doppler envelope. All echocardiographic Doppler measurements were performed by 2 experienced readers who were blinded to the hemodynamic data and to each other’s assessments (L.A. and A.S). Both readers analyzed all images independently. Discrepancies were resolved by consensus.

Acceleration time (AT) was measured in milliseconds as the time to peak velocity of the RVOT envelope measured from the beginning of ejection. At least 3 cardiac cycles were measured and averaged for analysis. Deceleration time was measured from the peak Doppler velocity to the end of ejection. Care was taken to align the sample volume and the axis of the bloodstream correctly to obtain the highest possible Doppler velocity signal.

RV size was measured as the basal RV dimension in the four-chamber RV-focused apical view. All other variables were obtained in accordance with the current American Society of Echocardiography guidelines.^21^ Multiple screenshots of 3 to 5 Doppler envelopes were obtained under optimal sweep, scale, and gain settings, to crisply define and capture notch morphology with minimal spectral dispersion. ESN pattern (spike and dome morphology) was visually assessed and deemed present if the Doppler envelope exhibited a narrow peaked initial wave with an early deceleration of the RVOT envelope producing a sharp notch within the first half of systole (ie, notch location within the initial 50% of ejection, as estimated using the online time caliper tool), followed by a lower amplitude signal (dome.) Similarly, MSN or LSN was defined as a distinct notch falling at the mid-point of the systolic ejection period or, if the nadir occurred closer to the end of ejection, respectively, dividing the flow profile into 2 distinct peaks. No notching referred to the absence of a discernible notch in the setting of a parabolic or triangular-shaped contour of the RVOT envelope.

**Figure 1:**
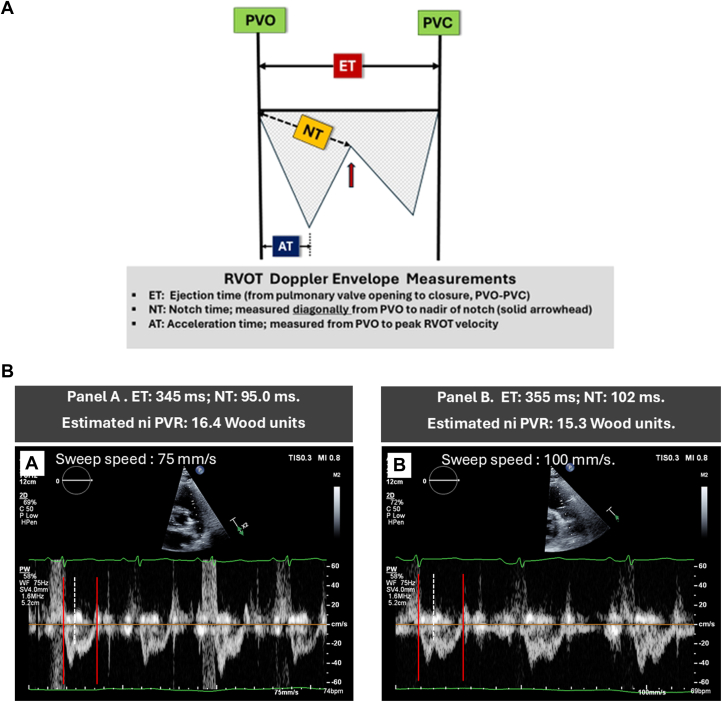
Schematic Illustrating RVOT Doppler Envelope and Technical Tips for Accurate Measurements A) The ratio: ET/NT is the core component of the Doppler Notch regression equation used to estimate niPVR. [niPVR = 7.1 · (Ejection time/Notch time) − 9.36]. (B) Case example of a 65 y/o with type 2 diabetes, COPD, emphysema, diastolic dysfunction, WHO type 3 pulmonary hypertension, and hypoxemic respiratory failure. Right heart cath data: pulmonary Artery (S/D/M): 101/41/65; pulmonary capillary wedge: 22/20/20; right atrium (a/v/M): 28/24/22 mm Hg (mean pressures bolded). Fick Cardiac output: 2.7 L/min. Estimated invasive pulmonary vascular resistance: 16.6 Wood units. See below estimations of niPVR obtained from RVOT Doppler envelopes at different sweep speeds (A and B) in the same patient. AT = acceleration time; COPD = chronic obstructive pulmonary disorder; ET = ejection time; niPVR = noninvasive pulmonary vascular resistance; NT = notch time; PVC = pulmonary valve closure; PVO = pulmonary valve opening; RVOT = right ventricular outflow tract.

Ejection time (ET) was measured in milliseconds from the beginning to the end of the RVOT Doppler envelope using online calipers. Notch time (NT) in milliseconds, representing the temporal width/duration of the Doppler notch was measured diagonally from the onset of ejection to the nadir of the notch (Figure 1A). This technique provided optimal accuracy and reproducibility for NT assessment, obviating the need for nadir extrapolation, otherwise required for estimation of NT horizontally. The ratio was then computed as ET divided by NT.

Notch morphology was best appreciated when the Doppler beam was aligned parallel to the RVOT outflow, with the pulse wave sample volume placed at the appropriate location, followed by scale, filter and baseline adjustments, acquired at sweep speeds of 50 to 100 mm (Figures 1A and 1B), at the discretion of the sonographer, to optimize notch definition.

### Statistics

The baseline data are expressed as medians (for non-normally distributed data) with range or means with SDs (for normally distributed data) for continuous variables and proportions for categorical variables. The normality of the candidate variables, NT, ET, acceleration time (AT), deceleration time, velocity of spike, velocity of dome, and RVOT VTI, were tested using skewness and kurtosis coefficients.

The agreement between the 2 authors was analyzed using the intraclass correlation coefficient with a 2-way random-effects model to measure the consistency of agreement and interobserver variability.

The derivation and validation cohorts were randomly selected from the final study cohort. Using the derivation cohort (n = 30), multiple backward stepwise linear regression analysis with its determination coefficient was used to determine the predictors of iPVR, beginning with all candidate variables (AT, deceleration time, NT, ET, RVOT VTI, spike velocity, and dome velocity). The choice of covariates for the regression was based on the plausibility that they could be associated with PVR. Collinearity was assessed using variance inflation factor analysis, and variables with high collinearity were excluded.

Using the estimated coefficients derived by the regression models, an equation that best predicted the iPVR was created and was labeled as the Doppler notch equation. The niPVR, derived from our Doppler notch equation and other previously published methods, were evaluated in our validation cohort against iPVR, the reference standard. The different equations compared include the following: Model 1: PVR = 10 × Peak TR velocity/RVOT VTI + 0.16 by Abbas et al^3^; Model 2: PVR= PASP/RVOT VTI + 3 by Opotowsky et al^22^; Model 3: PVR = 2.34 + TR PG/RVOT VTI × 1.48 by Kouzu et al^23^; Model 4: PVR = 5.19 × (TR velocity^2^/RVOT VTI) − 0. 4 by Abbas et al^4^, and Model 5: pulmonary vascular resistance index = 1.97 + 190.71 (systolic pulmonary artery pressure/[HR × RVOT VTI]) by Haddad et al^7^; and for model 5, the estimated pulmonary vascular resistance index was divided by body surface area to obtain PVR.

Correlations between different predictive models and iPVR were determined with correlation coefficient (r). The linear regression and correlation assumptions were verified by testing for heteroscedasticity using the White test and by examining the residual plots against the predictors. We also performed a Bland-Altman analysis with 95% confidence limits of agreement to compare the predictive performance of these published models with our model. Statistical analyses were performed using Stata software (version 18.0, StataCorp) and GraphPad Prism (version 10, GraphPad Software). The limit of significance was set as *P* ≤ 0.05.

## Results

A total of 95 patients referred for RHC (for suspected or known PH) between January 2020 and December 2023 who had an echocardiogram within 24 hours and met the inclusion criteria were screened for enrollment (Central Illustration).

**Central Illustration:**
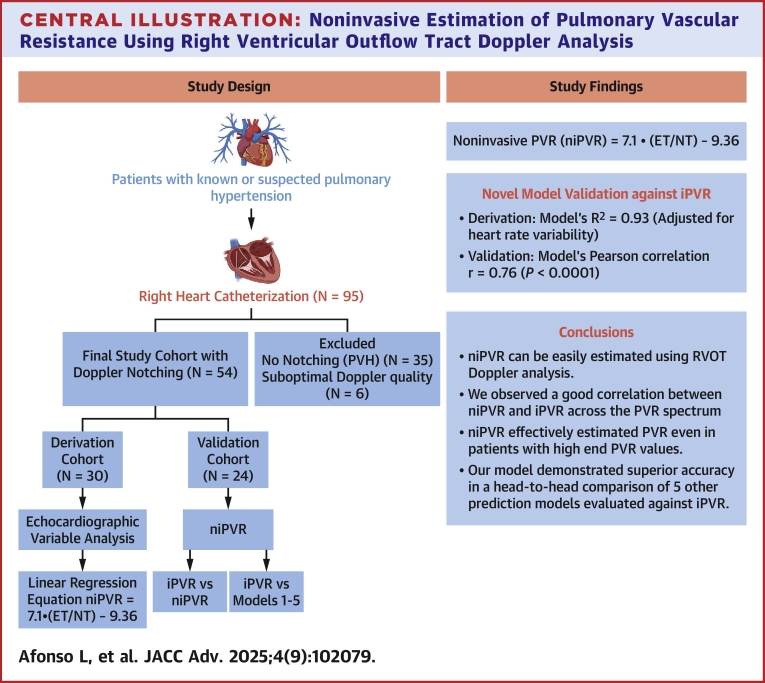
Noninvasive Estimation of Pulmonary Vascular Resistance Using Right Ventricular Outflow Tract Doppler Analysis ET = ejection time; IPVR = invasive estimation of PVR; mPAP = mean pulmonary artery pressure; niPVR = non-invasive estimation of PVR; NT = notch time; PVR = pulmonary vascular resistance; RVOT = right ventricular outflow tract.

In all, 35 patients (70.6% male, 62.1 ± 2.5 yrs), with IpcPH, based on invasive hemodynamics were excluded from the final PVR analyses, as they had no discernible Doppler notches and had RHC-derived PVR of <2.0 WU. Hemodynamic data in this cohort revealed the following: right atrial pressure: 14.3 ± 6.0 mm Hg; mean PAP: 34.6 ± 7.7 mm Hg; mean PCWP: 23.4 ± 6.4 mm Hg, and PVR: 1.71 ± 0.4 WU. The echocardiograms were notable for the absence of RVOT Doppler notching, triangular peaked Doppler envelopes, RVOT systolic Doppler AT: 95.6 ± 18.1 ms; RVOT-VTI: 10.4 ± 4.6 cm, and RV dilation (RV basal diameter: 4.4 ± 0.7 cm). Six other patients had to be excluded due to suboptimal/uninterpretable spectral Doppler envelope quality.

A total of 54 patients were included in the final PVR analyses. The demographics of the final study cohort are described in Table 1. Among these, 44% were included in the validation cohort. When compared to the derivation cohort, there were no significant differences in baseline characteristics between the 2 cohorts. Patients were predominantly females (57%), Black (72%) with heart failure (70%), an average age of 59.6 ± 16.4 years, and a body mass index of 30.1 ± 8.6. The most predominant comorbidities include hypertension (67%), diabetes (28%), coronary artery disease (31%), chronic obstructive pulmonary disease (35%), and smoking (43%).

**Table 1:** Demographic and Clinical Characteristics of the Final Study Cohort

	Total Cohort (N = 54)	Derivation Cohort (n = 30)	Validation Cohort (n = 24)	Standardized Mean Differences
Age (y)	59.7 (16.4)	60.2 (18.3)	58.5 (14.1)	0.10
Female, %	57.0	63.0	50.0	0.27
Race (AA) %	72.0	63.0	83.0	0.46
BMI (kg/m^2^)	30.1 (8.6)	29.2 (9.3)	31.3 (7.6)	0.25
BSA (Mosteller)	2.0 (0.3)	1.9 (0.3)	2.1 (0.4)	0.57
Systolic blood pressure	131.6 (28.5)	127.0 (20.5)	137.4 (35.7)	0.36
Diastolic blood pressure	82.0 (16.4)	79.1 (14.9)	85.6 (17.7)	0.40
Heart rate (bpm)	85.1 (12.7)	86.5 (13.2)	83.3 (12.0)	0.25
Hemoglobin (Hgb)	11.6 (2.5)	11.4 (2.5)	11.9 (2.6)	0.20
Hypertension	67%	60%	75%	0.33
Dyslipidemia	26%	17%	38%	0.48
Diabetes mellitus	28%	30%	25%	0.11
Chronic kidney disease	17%	17%	17%	0.00
End-stage renal disease	7%	7%	8%	0.04
Coronary artery disease	31%	27%	38%	0.24
Heart failure	70%	80%	58%	0.49
Chronic obstructive pulmonary disease	35%	30%	42%	0.25
Interstitial lung disease	17%	17%	17%	0.00
Sleep Apnea	26%	23%	29%	0.14
Current smoker	43%	50%	38%	0.24
HIV	4%	3%	4%	0.05
Cirrhosis/liver disease	9%	3%	17%	0.48
Connective tissue disease	4%	3%	4%	0.05
PH subgroups, n (%)				
Precapillary PH	24 (44.4%)	13 (43.3%)	11 (45.8%)	0.05
Combined precapillary and postcapillary PH	30 (55.6%)	17 (56.7%)	13 (54.2%)	0.05

Table 2 details invasive and echocardiographic characteristics across the derivation, validation, and total cohorts, with invasive metrics in total population showing a mean of 7.2 ± 3.1 WU for PVR with a wide range from 2.3 WU to 16.6 WU and a mPAP of 50.2 ± 11.4 mm Hg. Echocardiographic parameters show an average NT time was 129.5 ms with a total ET of 287.2 ms. The intraclass correlation coefficient between the 2 authors evaluating ET/NT for the validation cohort was 0.79 (95% CI: 0.56-0.90), indicating good reliability and highly significant agreement (F (31, 31) = 4.70, *P* < 0.001).

**Table 2:** Invasive and Echocardiographic Characteristics Across the Validation, Derivation, and Total Cohorts

	Total Cohort (N = 54)	Derivation Cohort (n = 30)	Validation Cohort (n = 24)	Standardized Mean Differences
Right heart catheterization data				
RA Mean (mm Hg)	16.3 (10.2)	15.0 (12.1)	17.9 (7.2)	0.29
PA (S) (mm Hg)	75.6 (18.7)	73.0 (19.0)	78.8 (18.3)	0.31
PA (D) (mm Hg)	35.2 (10.1)	32.3 (9.8)	38.6 (9.5)	0.65
PA Mean (mm Hg)	50.2 (11.4)	48.8 (11.7)	52.0 (11.0)	0.28
PCWP Mean (mm Hg)	18.9 (7.4)	17.0 (7.1)	21.3 (7.3)	0.60
TPG (mm Hg)	31.5 (12.0)	31.8 (12.2)	31.0 (12.0)	0.07
CO (assumed Fick; L/min)	4.5 (1.2)	4.4 (1.1)	4.6 (1.2)	0.17
Estimated PVR (WU)	7.2 (3.1)	7.4 (3.0)	7.0 (3.3)	0.13
PA Sat (%)	57.8 (9.2)	56.3 (9.7)	59.5 (8.4)	0.35
Art O_2_ Sat (%)	93.8 (5.2)	94.0 (4.3)	93.7 (6.0)	0.06
Echocardiography variables				
RVOT VTI (cm)	10.2 (3.4)	9.8 (3.0)	10.6 (3.8)	0.25
Acceleration time (msec)	65.6 (17.1)	64.8 (17.3)	75.6 (15.2)	0.66
Notch time (ms)	129.5 (28.9)	120.5 (19.9)	140.6 (34.4)	0.74
Ejection time (ms)	287.2 (37.3)	279.2 (32.1)	297.2 (41.5)	0.49
Ratio: ejection time/notch time	2.3 (0.4)	2.4 (0.4)	2.2 (0.4)	0.45
Sweep speed of RVOT Doppler (mm/s)	75.5 (22.2)	68.1 (18.1)	84.7 (23.7)	0.80
Tricuspid jet peak velocity (m/s)	3.6 (0.8)	3.6 (0.9)	3.6 (0.7)	0.07
RV basal diameter (cm)	5.1 (0.9)	5.0 (0.9)	5.2 (0.9)	0.24
Fractional area change (%)	23.8 (11.9)	21.5 (12.6)	28.1 (9.3)	0.59

Art O_2_ Sat = arterial oxygen saturation; CO = cardiac output; PA (D) = pulmonary artery diastolic; PA Mean = pulmonary artery mean; PA (S) = pulmonary artery systolic; PA Sat = pulmonary artery saturation; PCWP = pulmonary capillary wedge pressure; PVR = pulmonary vascular resistance; RA = right atrium; RVOT = right ventricular outflow tract; TPG = transpulmonary gradient; VTI = velocity time integral.

### Relationship between echocardiographic parameters and invasive pulmonary vascular resistance and derivation of the regression equation

Table 3 presents a stepwise linear regression analysis of variables (derivation cohort) used to predict iPVR. Initial models assessed individual predictors, such as AT and the inverse of NT, with corresponding R^2^ values indicating their predictive strength. As stated, multiple stepwise linear regression analysis defined the inverse of NT as the best predictor of iPVR with a determination coefficient (R^2^) of 0.53 (Table 3). This model significantly improved to a determination coefficient (R^2^) of 0.93 after adjusting for heart rate variability, using “ET”as a surrogate and represented in the Doppler notch regression equation as follows: niPVR = 7.1 · (ET/NT) − 9.36. Our Doppler notch equation model was not enhanced by the inclusion of additional variables (Table 3).

**Table 3:** Linear Regression Equations With Statistical Coefficient (R2) and Pvalues of Various Predictors of Invasive Pulmonary Vascular Resistance

Equation	Determination Coefficient *R*^2^	*P* Value
*α*_c_ = 0.85 + 0.02 · Et_*i*_	0.07	0.1702
*α*_c_ = 9.92 − 0.04 · Ac_*i*_	0.04	0.2445
*α*_c_ = 3.19 + 230.85 · invDt_*j*_	0.32	0.0013
*α*_c_ = 1448.18 · invNt_*i*_ − 4.87	0.53	<0.0001
*α*_c_ = 7.44 · invNt_*i*_ − 22 · invDt_*j*_ − 9.69	0.93	<0.0001
*α*_c_ = 7.13 · invNt_*i*_*/Et*_*i*_ − 9.36	0.93	<0.0001

This table presents a stepwise linear regression analysis of variables used to predict invasive pulmonary vascular resistance. Initial models assessed individual predictors, such as acceleration time and the inverse of notch time, with corresponding R^2^ values indicating their predictive strength. The final equation incorporates both inverse of notch time and ejection time, thus adjusting for heart rate variability and achieving the highest determination coefficient (R^2^ = 0.93). Each variable's significance and contribution to the model are detailed in the table.

α_c_ = PVR estimate; Ac_i_ = acceleration time; Et_i_ = ejection time; invNt_i_ = inverse of notch time; invDt_j_ = inverse of deceleration time (peak to base); Nt_j_ = notch time.

### Comparison of the performance of our prediction model to other published models in predicting invasive pulmonary vascular resistance using a validation cohort

**Figure 2:**
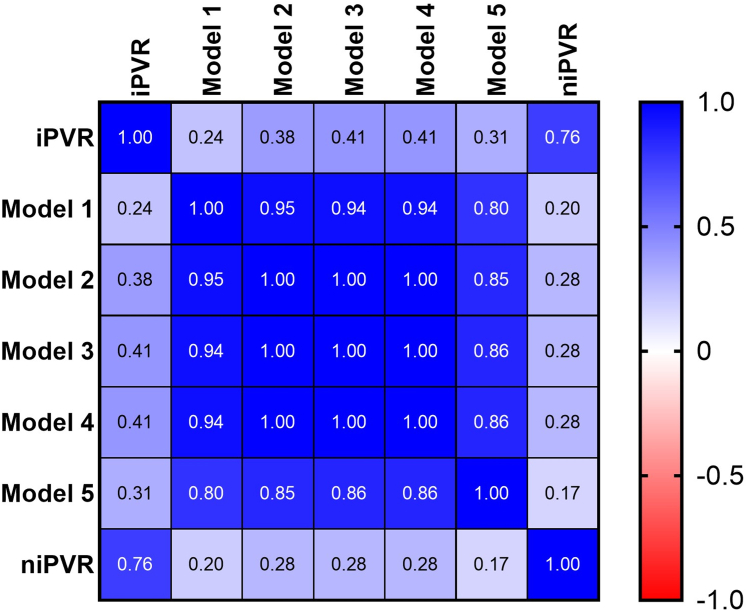
**Correlation Matrix Heatmap** The heatmap displays correlation coefficients between various variables, with values ranging from −1 (negative correlation, red) to 1 (positive correlation, blue). Darker shades represent stronger correlations. Abbreviations as in Figure 1.

**Figure 3:**
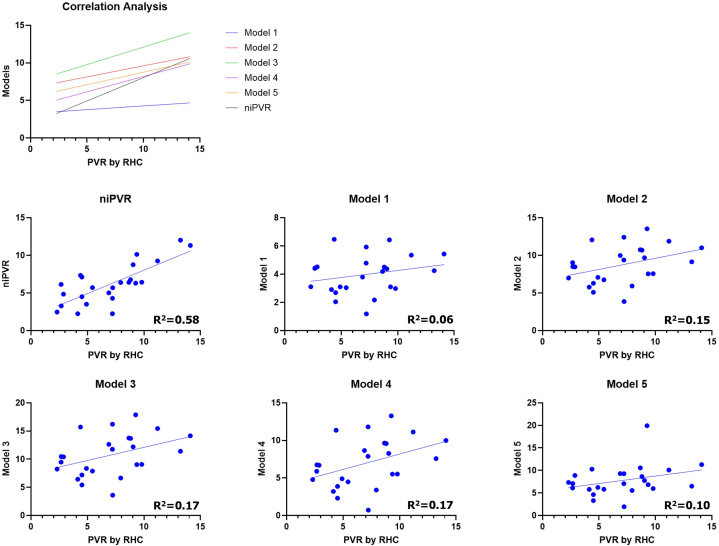
**Scatter Plots With Regression Lines** This figure presents scatter plots of models against iPVR. Each plot includes a regression line. The top panel displays the correlation trends for all models combined. PVR = pulmonary vascular resistance; RHC = right heart catheterization; other abbreviations as in Figure 1.

**Figure 4:**
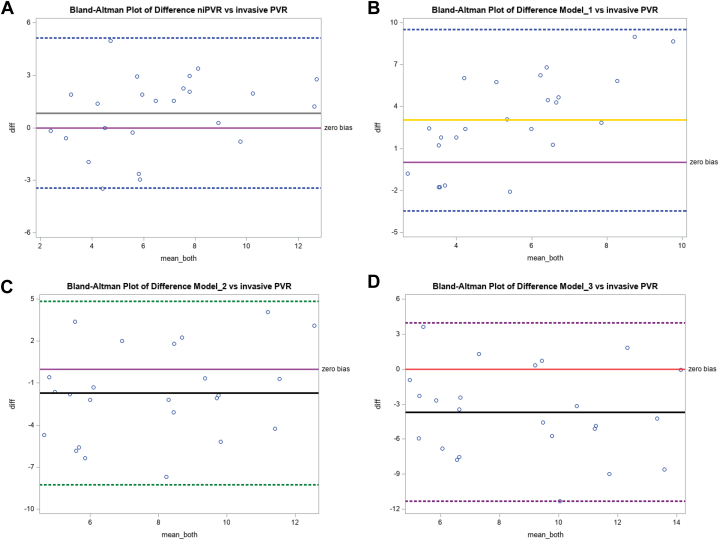

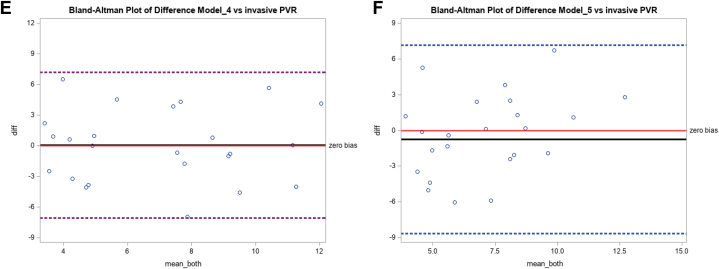
**Bland-Altman Correlation Plots** Bland-Altman Correlation plots show levels of agreement between the different models and invasive PVR. A) Doppler Notch Equation (niPVR) vs. iPVR. The paired t-test mean difference is 0.84 with a SD of 2.14 WU and *P* = 0.068 limit of agreement of 0.84 ± 4.19. Doppler Notch equation: niPVR = 7.1 · (Ejection time/Notch time) − 9.36 (Afonso et al); B) Model 1 vs. iPVR. The paired t-test mean difference is 3.05 with a SD of 3.24 WU and *P* = 0.0001 and limit of agreement of 3.05 ± 6.35. Model 1: PVR = 10 × Peak TR velocity/RVOT VTI + 0.16; Abbas et al^3^; C) Model 2 vs. iPVR. The paired t-test mean difference is (-) 1.71 with a SD of 3.28 WU and *P* = 0.018 and limit of agreement of (-) 1.71 ± 6.43. Model 2: PVR = PASP/RVOT VTI + 3, Opotowsky et al^22^; D) Model 3 vs. iPVR. The paired t-test mean difference is (-) 3.70 with a SD of 3.82 WU and *P* < 0.0001 and limit of agreement of (-) 3.70 ± 7.49. Model 3: PVR = 2.34 + TR PG/RVOT VTI × 1.48, Kouzu et al^23^; E) Model 4 vs. iPVR. The paired t-test mean difference is 0.05 with a SD of 3.56 WU and *P* = 0.949 and limit of agreement of 0.05 ± 7.00. Model 4: PVR = 5.19 × (TRV2 /RVOT VTI) − 0. 4, Abbas et al^4^; and F) Model 5 vs. iPVR. The paired t-test mean difference is (-) 0.75 with a SD of 3.96 WU and *P* = 0.360 and limit of agreement of (-) 0.75 ± 7.76. pulmonary vascular resistance index = 1.97 + 190.71 (systolic pulmonary artery pressure/[HR × RVOT VTI]), Haddad et al^7^; for model 5, the estimated pulmonary vascular resistance index was divided by BSA to obtain PVR. Abbreviation as Figures 1 and 3.

Our Doppler notch regression equation that best predicts iPVR (niPVR = 7.1 · [ET/NT] − 9.36) was validated in a separate validation cohort of patients and a comparison of the performance of our predictive model to other published models to predict iPVR was also performed in the same cohort. Figure 2 shows the Pearson correlation (*r*) between various models and iPVR (correlation matrix heatmap). The Pearson correlation coefficients and respective *P* values for the niPVR and other models compared with iPVR are presented in Table 4 as well as scatter plots in Figure 3. Our predictive model revealed the best correlation with a correlation coefficient (r) of 0.76 and *P* value of <0.0001 and model 1 having the worst correlation. Models 3 and 4 each showed moderate positive correlations with iPVR, (r = 0.41, *P* = 0.046), suggesting these models have moderate predictive capabilities. Although statistically significant, the correlations were marginal, indicating they just met the threshold for significance and accounted for only 16% of the variability in iPVR. Models 1, 2, and 5 did not reach the significance threshold and all had poor correlation with the iPVR. The Bland-Altman correlation plots (Figures 4A to 4F) were performed to assess the level of agreement between the different models and iPVR. The paired t-test mean difference between our model and iPVR was 0.84 with an SD of 2.14 WU and a *P* value of 0.068 (95% confidence limits of agreement, ±4.19) suggesting that this model provides the best estimate of iPVR when considering its level of disagreement compared to other models with limits of agreement of 3.05 ± 6.35, 1.71 ± 6.43, 3.70 ± 7.49, 0.05 ± 7.00, 0.75 ± 7.76 for Model 1, model 2, model 3, model 4, and model 5, respectively.

**Table 4:** Statistical Comparison of iPVR Vs Models 1-5 and niPVR (Doppler Notch Equation)

	iPVR vs Model 1	iPVR vs Model 2	iPVR vs Model 3	iPVR vs Model 4	iPVR vs Model 5	iPVR vs niPVR
r	0.24	0.38	0.41	0.41	0.32	0.76
95% CI	−0.18 to 0.59	−0.02 to 0.68	0.01 to 0.70	0.01 to 0.70	−0.10 to 0.64	0.52 to 0.89
R^2^	0.06	0.15	0.17	0.17	0.10	0.58
*P* Value	0.2635	0.0637	0.0431	0.0461	0.1341	<0.0001
*P* Value summary	ns	ns	a	a	ns	b

This table presents the correlation coefficients (r), R squared values, *P* values, and significance summaries for the comparison of pulmonary vascular resistance (PVR) measured by right heart catheterization (RHC) against 5 different models and noninvasive PVR (niPVR). Significance levels are noted as follows: ns (not significant). Model 1: PVR = 10 × Peak TR velocity/RVOT VTI + 0.16 by Abbas et al^3^; Model 2: PVR = PASP/RVOT VTI + 3 by Opotowsky et al^22^; Model 3: PVR = 2.34 + TR PG/RVOT VTI × 1.48 by Kouzu et al^23^; Model 4: PVR = 5.19 × (TRV^2^/RVOT VTI) − 0. 4 by Abbas et al^4^ and Model 5: pulmonary vascular resistance index = 1.97 + 190.71 (systolic pulmonary artery pressure/[HR × RVOT VTI]) by Haddad et al^7^ and for model 5, the estimated pulmonary vascular resistance index was divided by BSA to obtain PVR.

a (*P* < 0.05).

b (*P* < 0.0001).

### Visual qualitative assessment of Doppler envelope morphology

In addition to quantitative modeling, we observed that visual qualitative assessment of RVOT Doppler notching morphology may serve as a rapid approximation of PVR. Specifically, ESN was generally associated with PVR >5.0 WU, MSN corresponded to values around 5.0 WU, LSN was observed in the 3.0 to 5.0 WU range, and absent notching was typically associated with PVR <2.0 WU. These qualitative categories are supported by quantitative observations in our cohort. Specifically, we found that an ET/NT ratio >2 was typically associated with ESN and corresponded to elevated PVR values (>5.0 WU). An ET/NT ratio of approximately 2 was seen with MSN (PVR ∼5.0 WU), whereas a ratio <2 corresponded to LSN (PVR 3.0-5.0 WU). Patients with PVR <2.0 WU and no discernible notching were excluded from the analysis. These findings suggest that visual interpretation of RVOT Doppler morphology, when contextualized by timing metrics such as ET/NT, may offer a useful approximation of PVR in clinical practice, although further validation is warranted.

## Discussion

Measurement and/or estimation of PAP and PVR are the key to guiding therapeutic decisions in patients with PH. Although RHC, the current gold standard, is relatively simple and routinely performed, it remains invasive. Moreover, the need for repeat studies for follow-up or management adds to the associated costs and procedural risks. Accordingly, developing a robust noninvasive method to estimate PVR would not only be desirable but invaluable, and represents the central objective of this study.

Our study findings may be summarized as follows. 1) niPVR using RVOT Doppler notch analysis is feasible and easily estimated from routine echocardiograms performed for the workup of PH. 2) Our proposed prediction model (Doppler notch equation) offers a simple, rapid, quantitative, widely applicable method of noninvasively estimating PVR across the spectrum of abnormal PVR values. 3) The absence of distinct notching of the RVOT Doppler envelope identifies the subset of patients with pulmonary venous or isolated postcapillary hypertension (PVR <2.0 WU), reaffirming prior reports in the literature. 4) The correlation between iPVR and niPVR estimated using our methodology, is linear and remains robust, retaining high accuracy, even at the high end of the PVR spectrum, in sharp contrast to existing prediction models. 5) Our model provided the most accurate estimate of PVR, in a head-to-head comparison of 5 other published, echo-based prediction models, evaluated against iPVR.

Pressure and flow interactions are related to resistance in nonpulsatile systems and by impedance in pulsatile systems.^24^^,^^25^ Although resistance and impedance are used synonymously in the clinical arena, resistance represents a steady state parameter, expressed as a ratio of the mean arterial pressure drop across the pulmonary microvasculature to the cardiac output. Notably, PVR largely ignores the dynamic impact of opposition to pulsatile hydraulic flow ensuing from vascular stiffness or compliance.^26^ In contrast, impedance provides a composite measure of both static (PVR) and dynamic (pulmonary vascular stiffness) components of RV afterload. PVR is increased (>2.0 WU) in patients with pulmonary arterial or precapillary hypertension and low, in those with pulmonary venous or postcapillary hypertension (<2.0 WU). The normal pulmonary circulation exhibits a minimal amount of low-amplitude wave reflections that peak around the dicrotic notch. These insignificant reflections allow for optimal RV–PA coupling^27^ and the resulting laminar flow is represented by a parabolic RVOT Doppler envelope. Changes in impedance result in abnormal pressure wave reflections in systole, initially described in patients with primary PH.^28^ Reflected pressure waves are additive to incident pressure, whereas flow reflections subtract from forward flow, thereby augmenting RV afterload.^27^ The timing and magnitude of wave reflections are influenced by: 1) wave velocity (largely determined by vessel elasticity); 2) reflection distance (distance between site of ejection and site of reflection); and 3) reflection coefficient (the proportion of the antegrade propagating wavefront that is reflected).^14^^,^^29^ Although the main PA branches are major reflection sites, it is acknowledged that reflected waves from the pulmonary microcirculation may be equally important.^30^

Perturbations in flow patterns of right ventricular ejection have been previously described in canine experimental models^31^ and human studies of PH.^32^^,^^33^ Turkevich et al observed an inverse relationship (earlier partial systolic closing of the pulmonary valve on M-mode) with increasing severity of PH.^33^ Later, Torbicki et al. analyzed RVOT pulse wave Doppler recordings and observed an association between PAP and notched patterns, with shorter ATs characterizing patients with acute and chronic pulmonary embolisms.^32^ In the present study, we observed and leveraged the inverse relationship between NT and increasing PVR to derive the regression equation that was subsequently tested in our validation cohort.

Accompanying reductions in RVOT Doppler AT (<120 ms), strongly support the presence of PH, regardless of PH subtype. Our study findings also extend prior reports suggesting that the absence of Doppler notching is highly associated with pulmonary venous or IpcPH.^16^ The RVOT AT in the excluded IpcPH group (95.6 ± 18.1 ms), although shortened, was significantly longer than the measured AT in included groups of the precapillary hypertension group and the combined precapillary and postcapillary group (65.6 ± 17.1 ms). This intermediate value and the absence of Doppler notching reflects the distinct hemodynamics of IpcPH, where pulmonary venous congestion predominates without an accompanying increase in PVR.

Previous investigators have proposed noninvasive approaches of estimating PVR. Abbas et al. described methods based on the ratio of peak tricuspid regurgitant velocity to the (RV) outflow tract time velocity integral.^3^^,^^4^ Later, Haddad et al.^7^ attempted to incorporate PA systolic pressure and heart rate into their equation, claiming a stronger correlation of indexed PVR to invasive measurements of PVR. Although this method performed well at lower PVR values, the reported correlation coefficient at lower PVR values of 0.922 (*P* < 0.001), declined to 0.749 (*P* < 0.001) at higher PVR values.^7^ These approaches, although simple and appealing, remain collectively limited in their applicability, particularly at the high end of PVR values. Kouzu et al attempted to circumvent this limitation and derived their model from a population with elevated PVR; however, due to the universally high PVR in the derivation samples, their model includes a large constant, resulting in an overestimation of the PVR in the normal range.^23^ Scapellato et al. estimated PVR in a cohort of heart failure patients, using a ratio of systolic time interval involving the pre-ejection period, AcT, ET, and total systolic time; index = ([pre-ejection period/AcT]/total systolic time);^34^ however, this study did not include patients with PAH. More recently, Opotowski et al. explored the ratio of PASP Doppler to RVOT VTI and incorporated a constant, designating the presence of RVOT Doppler MSN, resulting in stronger agreement with catheterization estimates of PVR across a wide range of values.^22^

These preliminary data from a modest sample suggest that, among available echocardiographic methodologies for niPVR estimation, our model demonstrated closer correlation with iPVR across a wide range (2.3-14.2 WU) in the validation cohort. The disadvantage of existing methodologies, specifically, the poor correlation of niPVR with iPVR in the high-range of PVR, has been ascribed to progressive RV dysfunction and the development of right atrial hypertension leading to the unreliability of TR velocity as a surrogate measure of PA systolic pressure^6^ and the PA systolic pressure itself is a poor marker of progressive PH. Ventriculoarterial uncoupling ensues with progressive increases in afterload (PVR), leading to a loss of the linear relationship between PVR and mean or peak PAP (preserved coupling).^35^ These shortcomings prompted us to develop a TR jet velocity-independent metric of PVR, less influenced by RV performance, thus more reliable in the prediction of PVR in more advanced PH.

**Figure 5:**
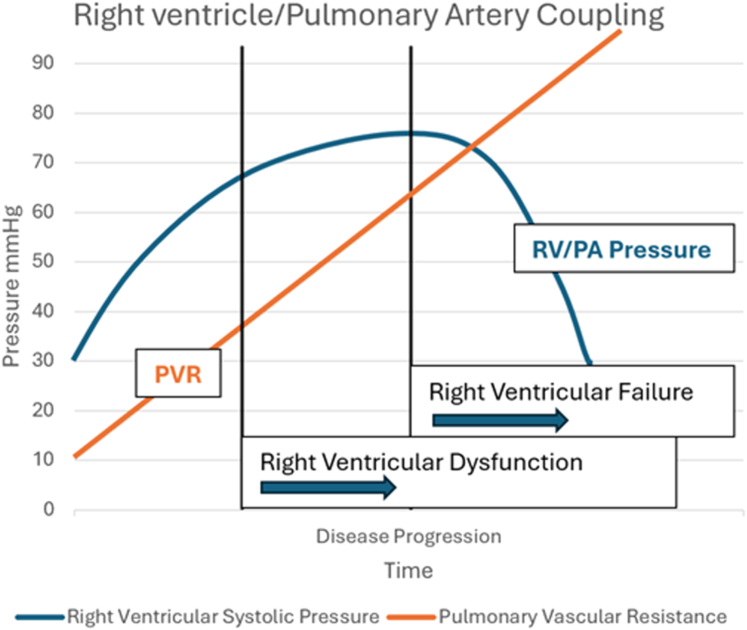
**Relationship Between PA Systolic Pressure, Pulmonary Vascular Resistance and Disease Progression** Schematic outlining relationship between PA systolic pressure, pulmonary vascular resistance and disease progression. PA = pulmonary arterial; RV = right ventricular; other abbreviation as Figure 3.

The ability to screen subtypes of PH (distinguishing precapillary from postcapillary hypertension) and predict PVR noninvasively is appealing for a variety of reasons. First, it would help with the early identification of patients (ie, those with Doppler notching) who would most benefit from RHC. Second, in patients with established PAH being treated with vasodilator pharmacotherapy, the estimation of PVR is better suited to monitor longitudinal response to therapy, as reciprocal changes in PVR and cardiac output, often lead to negligible changes in measured PA systolic pressure.^36^ Third, the progression of the disease is characterized by increasing right atrial pressure and PVR (again easily estimated using our prediction model), alongside declining cardiac output and PA systolic pressure (Figure 5).^37^

The presence and location of systolic notching can vary with respiration (varying RV stroke volume and translation), hypoxemia, Doppler misalignment, and optimal sample volume placement, nevertheless, based on our observations, best PVR correlations were obtained when cycles demonstrating earliest notching were selected for analysis. The incorporation of “ejection time” in our model introduces a correction for heart rate, allowing rate-adjusted comparisons of niPVR estimates in serial exams and between patients. The estimation of PAP and PVR, using existing models is often challenging and hindered by misaligned, absent/suboptimal TR Doppler signal quality. Importantly, our model provides the ability to estimate PVR independent of the presence or absence of tricuspid regurgitation.

### Study Limitations

A limitation of our study was the small number of patients enrolled but all studied patients had RHC and echocardiography performed within 24 hours of each other. Another potential limitation of our study is the use of the assumed Fick equation with its inherent assumptions, and measurement errors of oxygen consumption; however, this method has been lately the standard of care in laboratories for performing RHC. Yet another limitation of our notch-based method might be related to a beat-to-beat variation in notch configuration or notch expression, seen occasionally in some patients, a finding elegantly demonstrated by Kitabatake et al.^15^ in the setting of atrial fibrillation. Using simultaneous recordings of RV flow velocity, RV and PA hemodynamic pressure tracings, these investigators verified this phenomenon and mechanistically attributed it to low RV stroke volume (low pulse pressure), apparent in beats when peak right ventricular pressure did not exceed peak PAP. Accordingly, the accuracy of our methodology in patients with underlying atrial fibrillation or those with very advanced RV systolic dysfunction and low RV stroke volume will need further study. It is important to note that our methodology is applicable only to patients with clearly discernible RVOT notching. Not all individuals with elevated PVR will exhibit notching, which may be attributable to physiologic factors—such as reduced effective RV stroke volume in the setting of severe tricuspid regurgitation—patient selection or technical limitations related to Doppler signal acquisition Although our proposed technique may minimize the need for, reduce the risk of radiation exposure, and potential complications associated with RHC, it should by no means be viewed as a replacement for RHC.

In summary, this was a single-center, proof-of-concept study performed at a tertiary-care center in a modest cohort of patients, our findings will need further prospective external validation in the general population with PH (precapillary and postcapillary PH) to evaluate the robustness and generalizability of our results.

## Conclusions

Noninvasive estimation of PVR using the proposed Doppler notch equation is feasible, correlates well with invasively obtained measurements and is highly reproducible in patients with precapillary or combined precapillary and postcapillary PH.

Perspectives **COMPETENCY IN PATIENT CARE AND PROCEDURAL SKILLS:** This Doppler-based method for estimating PVR is practical across diverse clinical settings, including those without access to RHC. Clinically, it enables accurate, noninvasive PVR assessment in advanced PAH—even without tricuspid regurgitation—supporting diagnosis, risk stratification, and reducing reliance on invasive testing. Procedurally, it promotes proficiency in RVOT Doppler waveform analysis and recognition of systolic notching, advancing core echocardiographic skills aligned with patient-centered cardiovascular care.

**TRANSLATIONAL OUTLOOK:** This approach offers a physiologically grounded, scalable tool for noninvasive PVR estimation. It has potential for integration into clinical or AI algorithms, longitudinal monitoring, and predictive models when combined with clinical and biomarker data. Prospective multicenter studies are needed to validate its role as a prognostic and therapeutic response marker across PAH subtypes and platforms.

## Funding support and author disclosures

The authors have reported that they have no relationships relevant to the contents of this paper to disclose.
